# Tumor relevant protein functional interactions identified using bipartite graph analyses

**DOI:** 10.1038/s41598-021-00879-2

**Published:** 2021-11-02

**Authors:** Divya Lakshmi Venkatraman, Deepshika Pulimamidi, Harsh G. Shukla, Shubhada R. Hegde

**Affiliations:** Institute of Bioinformatics and Applied Biotechnology (IBAB), Bengaluru, 560 100 India

**Keywords:** Cancer, Computational biology and bioinformatics, Systems biology

## Abstract

An increased surge of -omics data for the diseases such as cancer allows for deriving insights into the affiliated protein interactions. We used bipartite network principles to build protein functional associations of the differentially regulated genes in 18 cancer types. This approach allowed us to combine expression data to functional associations in many cancers simultaneously. Further, graph centrality measures suggested the importance of upregulated genes such as BIRC5, UBE2C, BUB1B, KIF20A and PTH1R in cancer. Pathway analysis of the high centrality network nodes suggested the importance of the upregulation of cell cycle and replication associated proteins in cancer. Some of the downregulated high centrality proteins include actins, myosins and ATPase subunits. Among the transcription factors, mini-chromosome maintenance proteins (MCMs) and E2F family proteins appeared prominently in regulating many differentially regulated genes. The projected unipartite networks of the up and downregulated genes were comprised of 37,411 and 41,756 interactions, respectively. The conclusions obtained by collating these interactions revealed pan-cancer as well as subtype specific protein complexes and clusters. Therefore, we demonstrate that incorporating expression data from multiple cancers into bipartite graphs validates existing cancer associated mechanisms as well as directs to novel interactions and pathways.

## Introduction

Cancer is proving to be one of the deadliest diseases, with 18 million new cases and 9.6 million deaths in 2018^1^. This invites attention for research, diagnosis and treatment of cancer. Cure for cancer is elusive due to the heterogeneity and complexity of its manifestation^2^. Decades of research on cancer has accumulated information regarding histopathology, gene expression, signaling events and protein interactions (reviewed in Ref.^3,4^). For long, cancer studies were largely focused on the molecular level. With the emergence of systems biological approaches, information such as gene expression and pathway functioning are integrated to generate holistic perspective for the diseases such as cancer^5^.

One of the efficient ways to understand the difference between cancer cells and their normal counterparts is to study their differential gene expression signatures. The large-scale expression data for cancer and normal samples derived from the RNA-seq experiments available in multiple databases facilitate such system-wide analyses. Apart from studying individual cancer types, data from multiple cancers could be combined to understand common principles governing disease establishment and their inter-connectedness. This is useful in distinguishing pathways and proteins which are unique to a given cancer from the ones that are shared by multiple cancers. Pan-Cancer analysis project is one such attempt by TCGA (The Cancer Genome Atlas) to characterize cancers according to their molecular and genetic profiles^6^. Earlier, DNA microarray data spanning multiple cancers was analysed to obtain functional modules of genes showing expression or repression for a given cancer^7^. Also, microarray-based gene expression data from multiple cancers were considered to identify functional pathways that are dysregulated across various cancer types^8,9^. Along similar lines, RNA-seq data based study reported differential gene expression signatures across 33 human cancer types as well as between highly and lowly-advanced cancers^10^. The differential gene expression information was also mapped onto known protein complexes to establish connections between cancers and their corresponding enriched protein complexes^11^. In another study, systematic cataloging of somatic mutations was performed to obtain mutational signatures in many different cancers^12^. Likewise, in a pan-cancer mode, somatic copy number variations were analysed to identify their common patterns of occurrence in cancer^13^. Over the years, such studies proved widely informative in establishing relatedness between different cancers. Besides these, it is useful to view the differentially regulated genes as functionally interacting proteins based on their expression pattern in multiple cancers.

The cancer specific association between genes and the pathways can be aptly envisioned in the form of interaction networks (graphs). Such interaction networks provide convenient ways to contextualize several biomolecules by describing their mode of association^14^. While protein–protein interactions are represented as an undirected graph, directionality is attributed from one node (source) to another (target) in the gene regulatory networks, cell signaling networks and phosphorylation networks^15^. Bipartite networks, on the other hand, represent interactions between two sets of nodes where the connections run only across the sets^16^. Many relationships in the real-world are modeled as bipartite graphs. For instance, a bipartite network of scientific collaborations includes scientists and their research papers, actor collaboration network includes actors and the movies they have worked in and the human disease network associates genes and the diseases^17–19^. Bipartite graphs can be converted into one-mode projections and further analysed using graph theoretical methods^20^. One mode projection of a bipartite graph contains nodes belonging to one set, and the edges are drawn between them based on their connections to the nodes of the other set in the original bipartite graph. For example, in the human disease network, two diseases are connected if they share many common genes involved in the disease. Similarly, two genes are connected if they tend to be associated with the same disease^19^.

Recently, several computational and statistical approaches for defining driver genes based on cancer genomics data have been suggested^21^. One such approach is the network-based method which greatly increases the precision of predicting driver genes^22^. Bipartite graph analysis has been widely used to predict the links between tumor samples and genes, and also to identify cancer driver genes of various individual cancers such as breast, lung and prostate^22,23^. We performed bipartite network analysis of the differentially expressed genes in multiple cancer types to identify protein functional associations that are pertinent to cancer. Graph theoretical analyses—including centrality, clusters and motifs of the derived networks revealed important proteins and pathways associated with cancer. Our study, therefore, emphasizes on how the graph theoretical method, namely bipartite network analysis, can be effectively used in cancer systems biology to integrate the emerging large-scale data.

## Results

### Identifying differentially expressed genes across multiple cancer types

**Figure 1:**
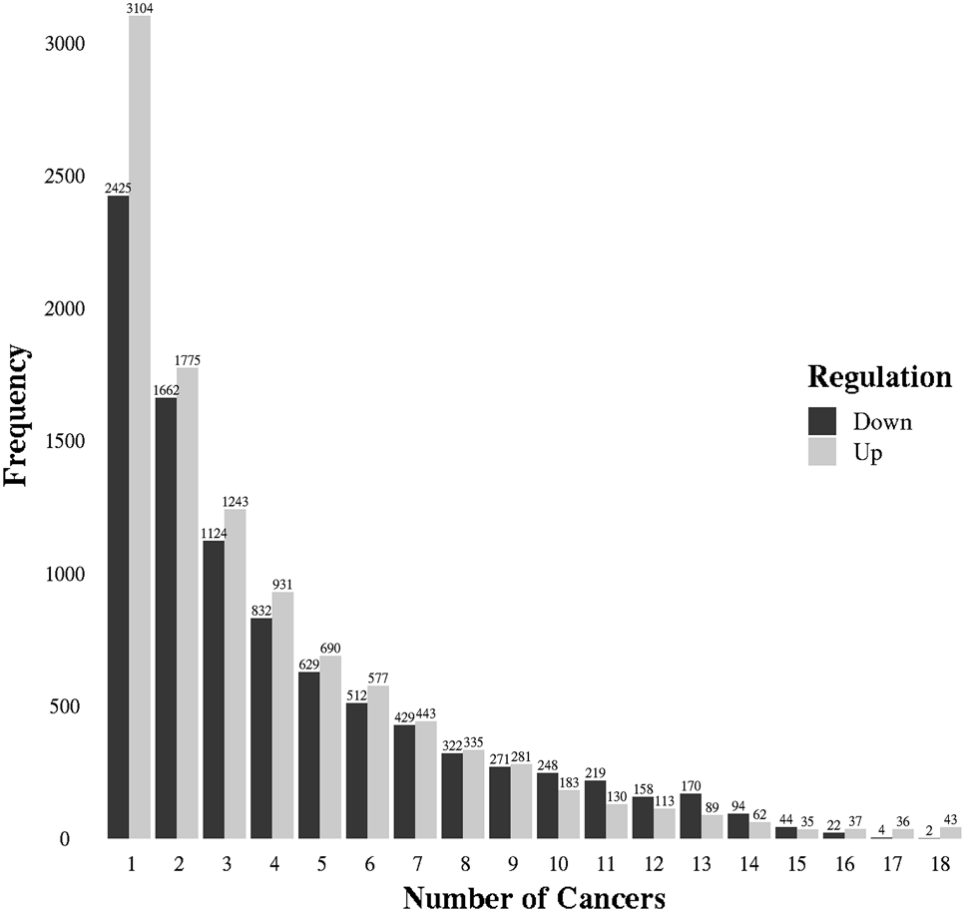
Bar plot representing the frequency of the differentially expressed genes against number of cancers. There are many genes which appear as differentially regulated in one or a few cancer types. However, comparatively smaller set of genes are differentially regulated in many cancer types.

We used RNA-seq data corresponding to 18 cancer types along with their control samples to obtain differential gene expression^24^. There were 10,107 and 9167 genes identified as up and downregulated in one or more cancers, respectively, with an overlap of 5333 genes (Table 1, Supplementary Table S1). Of these, many genes were differentially expressed in only one or a few cancers while some were differentially expressed across many cancer types (Fig. 1). For example, Parathyroid Hormone 1 Receptor (PTH1R) and collagen-binding Dermatopontin (DPT) were downregulated in all the 18 cancers. Previously, decreased DPT expression was seen in uterine leiomyomas^25^. Also, expression of the PTH1R was significantly reduced in hepatocellular carcinoma compared to normal liver tissues^26^. In line with our observations, the downregulation of PTH1R and DPT could be important for most of the cancers. Also, 43 genes showed upregulation in all the 18 cancers. These include cell cycle associated genes CCNB2, CDC45, BUB1B, TTK, CDC25C, PKMYT1 and BUB1 (KEGG pathway enrichment with adjusted P-value < 2.4.9e−06)^27^. Further, we compared these with the previous literature to highlight some of the known upregulated genes in cancer. BIRC5 which codes for survivin protein is highly expressed in almost all the human cancers^28^. Survivin is also considered as a potential target for tumor therapy^29^. Another upregulated protein ubiquitin-conjugating enzymes 2C (UBE2C) is involved in the degradation of mitotic cyclins such as cyclin B and facilitates cell cycle progression. Elevated expression of UBE2C also correlates with poor survival and increased risk for relapse^30^. A member of the serine/threonine kinase protein family NEK2 also shows overexpression in multiple cancer types which is indicative of relapse and poor survival^31^. Some of the other upregulated genes include centromere proteins (CENPA, CENPF and CENPM), kinesin family members (KIF20A and KIF4A), DNA replication factor CDT1, holliday junction recognition protein HJURP, and G2 and S phase-expressed protein GTSE1. Pan-cancer expression analysis, therefore, suggests the importance of these differentially expressed genes in cancer.

**Table 1:** List of cancer types included in the study along with the number of normal and tumor samples and their differentially regulated genes.

Cancer type	Normal samples	Tumor samples	Genes upregulated	Genes downregulated
Bladder urothelial carcinoma (BLCA)	28	362	1724	1390
Breast invasive carcinoma (BRCA)	322	982	2409	2305
Cervical and endocervical cancers (CESC)	13	259	2529	2030
Colon adenocarcinoma (COAD)	380	285	2292	3260
Esophageal carcinoma (ESCA)	670	183	2885	2359
Head and Neck squamous cell carcinoma (HNSC)	97	460	1593	2605
Kidney renal clear cell carcinoma (KIRC)	104	475	1780	1389
Liver hepatocellular carcinoma (LIHC)	163	295	1581	912
Lung adenocarcinoma (LUAD)	372	503	2502	2040
Prostate adenocarcinoma (PRAD)	154	426	963	2220
Stomach adenocarcinoma (STAD)	225	380	1984	1943
Thyroid carcinoma (THCA)	371	441	1372	2135
Uterine Corpus Endometrial Carcinoma (UCEC)	105	141	3411	2409
Cholangiocarcinoma (CHOL)	9	31	2680	1566
Kidney Chromophobe (KICH)	25	60	1388	1909
Kidney renal papillary cell carcinoma (KIRP)	29	236	1353	1162
Lung squamous cell carcinoma (LUSC)	51	489	3338	2657
Rectum adenocarcinoma (READ)	10	87	2667	3807

### High centrality nodes in the cancer associated bipartite networks

In order to connect the cancer types and their differentially regulated genes, we built two cancer-gene association networks representing the up and the downregulated genes. Differentially regulated genes were modeled as bipartite graphs BG = (C, G, E), where C is the set of nodes representing a cancer type, G is the set of nodes representing differentially expressed genes and E is the set of edges in the graph.

Further, we tested whether the distribution of the node redundancy coefficients in the real networks are different than the distribution in random networks with similar degrees. For this, 10 random bipartite networks with degree distributions similar to the real network were created using a configuration model, thereby ensuring that the scale free property of the generated random networks is preserved. These random networks and the two real networks representing up and downregulated genes in cancer were compared using two-sample Kolmogorov Smirnov test for the distributions of the node redundancy coefficients (“Methods”). For the upregulated bipartite network, the average D-statistic of the node redundancy coefficient was 0.833 (P-value < 1.5E−06). Similarly, for the downregulated bipartite network, the average D-statistic of the node redundancy coefficient was 0.788 (P-value < 1.3E−05). Significantly high D-statistic for node redundancy in each of these comparisons suggested that the generated bipartite networks for the up and downregulated genes in cancer were indeed different from the random networks, thus implying their biological significance. For these bipartite graphs, topological indexes such as centralities were calculated which score the nodes by estimating their importance in the network^32^. Degree centrality in a bipartite measures the number of connections a node makes in the opposite node set. Therefore, genes which are up or downregulated in multiple cancers are evaluated as important by the degree centrality measure. Closeness centrality evaluates how rapidly a given node can communicate with the other nodes in a network through short paths. In a bipartite graph, a node can have a minimum distance of 1 to all nodes of the other set and a minimum distance of 2 from all nodes of its own set. Likewise, high betweenness centrality in a bipartite network implicates nodes that mediate communication between other pairs of nodes via multiple shortest paths crossing through them. Therefore, these centrality measures provide diverse aspects of node importance in the bipartite network. We aggregated the top 5% nodes of the betweenness, closeness and degree centrality measures, which yielded 616 and 619 genes for the downregulated and upregulated networks, respectively, without any overlap (Supplementary Table S2). We observe that 66% (407 out of 619) and 51% (313 out of 616) of the top 5% aggregates are evaluated as important by all the three centrality measures for the up and downregulated networks, respectively. A close inspection of these individual centrality measures revealed that while top 5% of degree centrality results were nodes with a minimum degree of 11 for the upregulated and 12 for the downregulated network, top 5% of the closeness and the betweenness centrality measures shortlisted some of the nodes which were differentially regulated even in 8 cancer types (Supplementary Fig. S1). Hence, we considered these genes as significant for further analyses.

**Figure 2:**
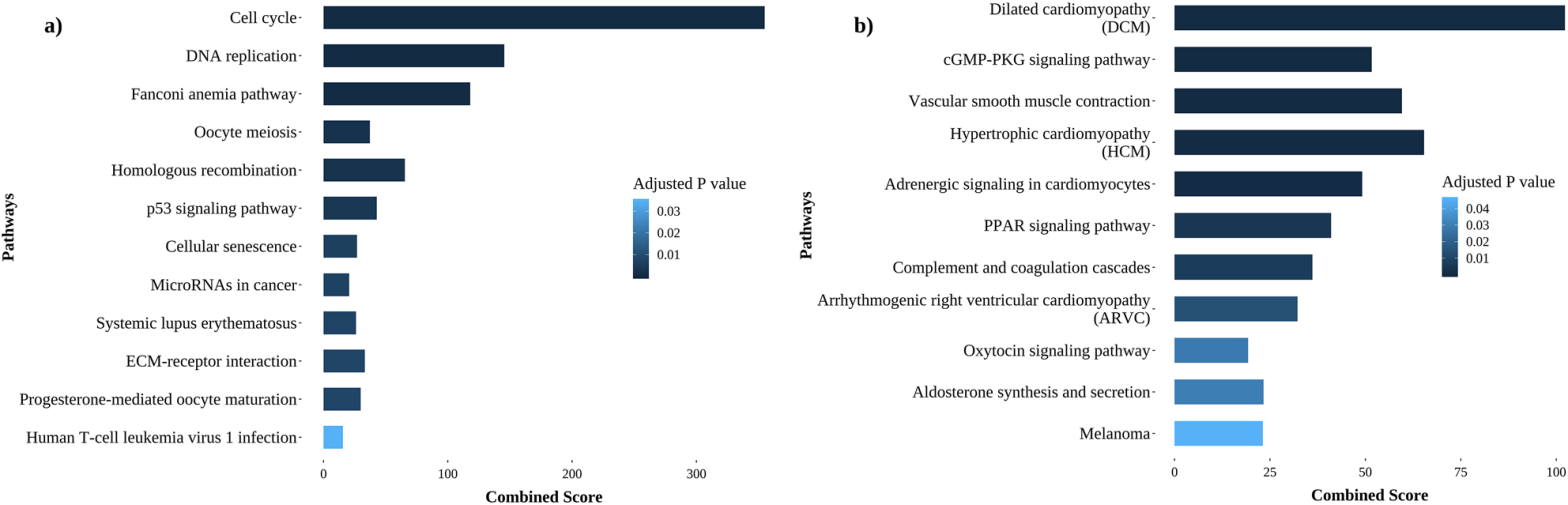
Bar plots representing the enriched pathways of the top 5% high centrality nodes (Adjusted P-value versus Combined score). Combined score represents the product of logarithmic P-value computed using Fisher exact test and z-score. These scores for each pathway are obtained from Enrichr (gene enrichment analysis web-based tool). (**a**) Upregulated, (**b**) downregulated.

Pathway enrichment of the high centrality genes in both up and downregulated bipartite networks was performed using Enrichr (KEGG pathway enrichment with adjusted P-value < 0.05)^27^. Cell cycle, Fanconi anemia pathway, DNA replication, homologous recombination and P53 signaling are some of the prominent pathways represented by the upregulated high centrality nodes (Fig. 2a). On the other hand, Dilated cardiomyopathy, Vascular smooth muscle contraction, Hypertrophic cardiomyopathy, Adrenergic signaling in cardiomyocytes and Arrhythmogenic right ventricular cardiomyopathy pathways were enriched for the downregulated high centrality nodes (Fig. 2b). The downregulated proteins from these pathways included actins (ACTC1, ACTG2), myosins (MYH11, MYH7, MYLK, MYL3, MYL9), protein phosphatase 1 regulatory subunits (PPP1R12B, PPP1R12C, PPP1R14A, PPP1R1A), microRNA protein coding genes (CTNNA3, DMD, MYH7, PDE2A), complement proteins (C1R, C6, C7, CFD) and ATPases (ATP1A2, ATP1B2). Apart from their primary functions such as muscle contraction, motility process, transport and metabolism, these genes also participate invarious other cellular processes. For example, actins are involved in cell division, migration and signaling, myosins are associated with cell migration and adhesion, and protein phosphatase 1 regulatory subunits are implicated in cell cycle progression, protein synthesis, transcription and neuronal signaling^33–35^. Importantly, few of these proteins, namely, ATP1B2, CTNNA3 and MYLK were reported to be downregulated biomarkers in glioma, hepatocellular and breast carcinoma, respectively^36–38^. Therefore, it appears important to investigate the functional implications associated with the downregulation of these proteins in cancers.

The nodes which we identified as central in cancer associated bipartite graphs were either up or downregulated in multiple cancers. Such a behavior is governed by the transcription factors which act as activators and/or repressors of gene expression. We tested the transcription factor enrichment for the 1235 differentially regulated high centrality genes. For further analysis, we considered top 25% enriched transcription factors with adjusted P-value < 0.05 which regulate 10 or more high centrality genes. The complete list of regulatory interactions is given in Supplementary Table S3. We observe that the mini-chromosome maintenance proteins (MCMs) MCM2, MCM3, MCM6 and MCM7, which were upregulated in most of the cancers, also regulate the expression of a significant number of high centrality genes in the network. In addition, many high centrality differentially regulated genes in cancer were regulated by the E2F family genes, of which E2F4 alone regulates about 344 genes. E2F1, E2F2 and E2F7 proteins, which were upregulated in most of the cancer types and appear as high centrality nodes in the bipartite graphs, regulate around 224, 93 and 74 high centrality genes, respectively (Supplementary Table S3). As E2F family transcription factors participate in cell cycle control, the overexpression of E2F family genes and their subsequent gene regulatory activities might have significant implications on cancer cell proliferation. We also observed that transcription factors E2F4 and ETS1 regulate 41 and 38 of the 43 genes which were upregulated in all the cancers, respectively (Supplementary Tables S1 and S3). Some of the other enriched transcription factors include MYB, BRCA1, STAT1 and ETV4 which are upregulated in majority of the cancer types. On the other hand, Retinoid X receptor RXRG which is a regulator of 15 high centrality differentially regulated genes is downregulated in 12 cancer types. Earlier in an ovarian adenocarcinoma progression model, activation of RXRG by retinoid treatment sensitized the cells to apoptosis^39^. Further analysis of the genes which are differentially expressed in most of the cancers and also regulate many high centrality nodes in the network could provide more insights on the common principles governing tumor development.

### Weighted one-mode projection of the cancer associated bipartite networks

Depending on the nature of expression across different cancer types considered, we derived functional association between differentially regulated genes in the original bipartite networks. The one-mode projection of a bipartite graph results in two sets of unipartite graphs in which two nodes of G (or C) are connected if they have at least one edge in common in the original bipartite graph (Supplementary Fig. S2). To obtain cancer specific protein functional interactions, we projected the bipartite graphs representing up and downregulated genes onto their respective unipartite graphs. While projecting, each edge in these graphs were weighed using a Jaccard index to account for the similarity in association (“Methods”). The distribution of the number of components and the size of the largest component at various weight-cutoff are provided in Supplementary Fig. S3. In order to eliminate insignificant edges, we used 0.9 as the edge weight cutoff and analyzed the resulting protein functional interaction networks. These comprised of 37,411 edges among 4544 nodes and 41,756 edges among 4584 nodes for the up and the downregulated genes, respectively (Supplementary Table S4). While many of these interacting proteins are differentially regulated in majority of the cancer types, some of them seem to be specific for the known cancer subtypes^40^.

There was an overlap of 479 interactions (P-value < 2.50E−162) among 363 proteins between the upregulated projected network and the curated protein–protein interaction network (Supplementary Table S5a). These include extensive interactions among proteins AURKB, BUB1, BUB1B, BIRC5, CCNB2, CDK1, CDCA5, CDCA8, CENPE, CENPM, CENPI, CENPH, CENPA, ERCC6L, NDC80, NUF2, SKA1, SPC24, and SPC25 (Supplementary Table S5a). Also, there were 67 known interactions between 112 (P-value < 7.43E−03) proteins in the downregulated projected network. While some of these proteins such as SNRNP70 and SRSF5 are the components of the spliceosome complex, the others, namely, SORBS1, TPM2 and MYH11 were reported to have a role in tumor metastasis and development (Supplementary Table S5a)^41–43^. These up and downregulated proteins were differentially expressed in more than 8 cancer types studied. Additionally, the projected upregulated network had 77 known regulatory interactions of the transcription factors including E2F1, E2F7, MCM2 and MCM4 (Supplementary Table S5b). The downregulated projected network had 62 known regulatory interactions, of which transcription factors TAL1, ZEB1 and MEIS1 regulate the genes which were differentially regulated in more than half of the cancers studied (Supplementary Table S5b). The upregulated transcription factors E2F1, E2F7, MCM2 and MCM4, as previously mentioned, belong to the group of 25% high centrality nodes and therefore their activities might be important for cancer development.

**Figure 3:**
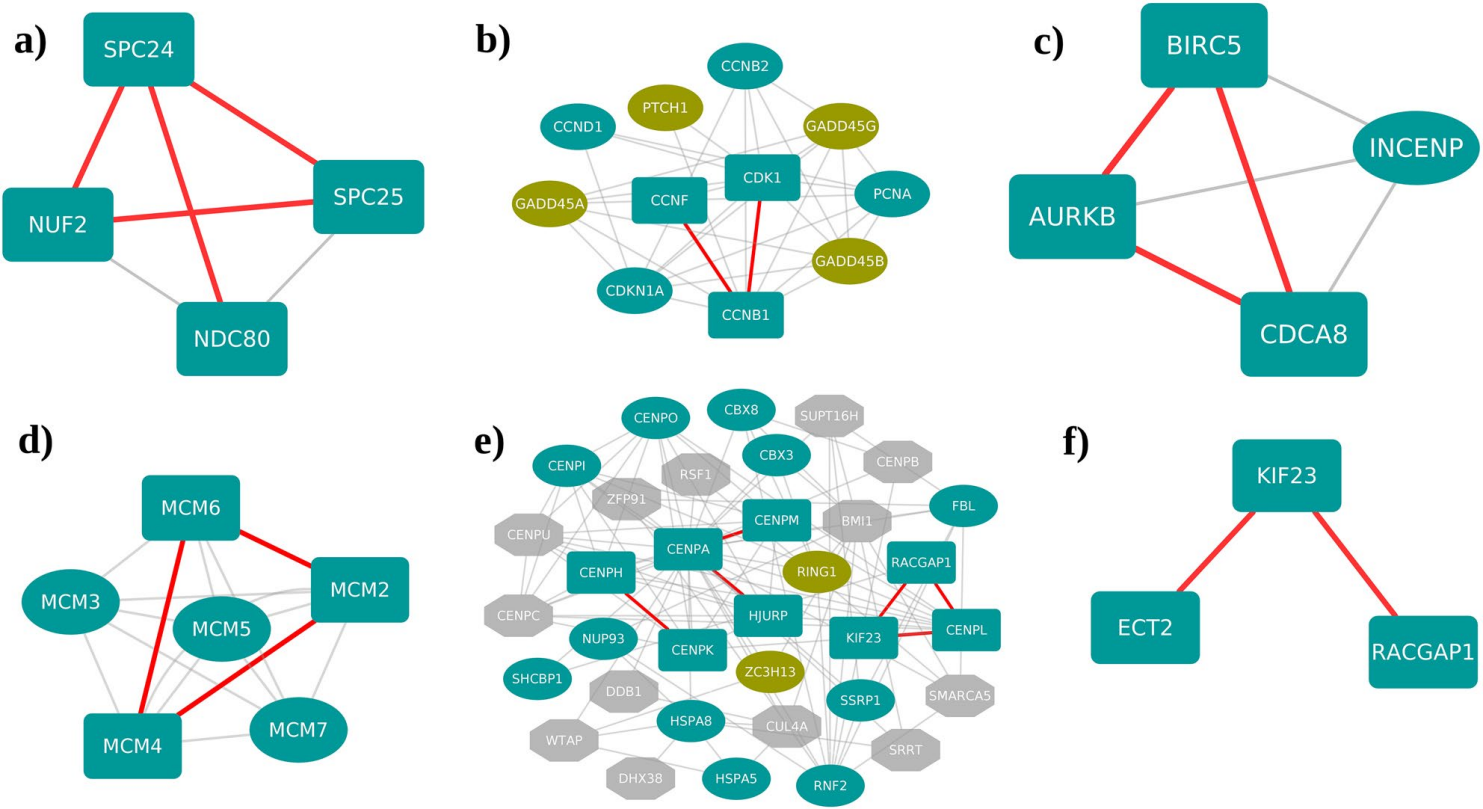
Interactions of the projected upregulated protein complexes. Rectangle shaped nodes are part of the upregulated projected network and the ellipse shaped nodes are differentially regulated components of the complex. Hexagons—other components of the complex. The red colored edges are the captured interactions between complex components in the projected network. (Blue nodes—upregulated, green nodes—downregulated and grey nodes – not differentially regulated) (**a**) NDC80 kinetochore complex, (**b**) cell cycle kinase complex, (**c**) chromosomal passenger complex (CPC), (**d**) mini-chromosome maintenance (MCM) protein complex, (**e**) Centromere (CEN) complex and (**f**) ECT2–KIF23–RACGAP1 complex.

The largest component of the up and downregulated network consisted of 6498 edges between 564 nodes and 9984 edges between 850 nodes, respectively. We observed that the genes which were part of these largest components were differentially regulated in 9 or more cancer types. These were compared with 4274 protein complexes derived from CORUM database to verify if we captured any known protein complexes. There were 14 protein complexes which showed overlapping interactions with the largest components of the projected networks. Some of the complexes found in the upregulated projected networks are shown in Fig. 3. NDC80 kinetochore complex, which plays a key role in chromosome alignment and segregation during mitosis, comprises of 4 components namely, NDC80, NUF2, SPC24 and SPC25. Out of the 6 possible interactions among these components, we captured 4 interactions in our network (Fig. 3a). NDC80 Kinetochore complex was previously reported to be involved in various types of cancers, including prostate and breast cancer^44,45^. In addition to the interactions between cell cycle kinase complex proteins CCNB1, CDK1 and CCNF (Fig. 3b), interactions of chromosomal passenger complex components AURKB, BIRC5 and CDCA5 were also recorded (Fig. 3c). Chromosomal passenger complex regulates cell division and has been implicated in breast cancer^46^. The MCM complex which plays a major role in cellular development and cell cycle consists of 6 components MCM2–MCM7, of which MCM2, MCM4 and MCM6 were found to interact in the projected upregulated network (Fig. 3d). Also, interactions between the proteins CENPA, CENPM, CENPK, CENPH and HJURP were identified which belong to centromere complex that regulates the kinetochore and spindle assembly (Fig. 3e). The network consisted of interactions among ECT2, KIF23 and RACGAP1 which belong to a complex associated with cytokinesis function (Fig. 3f). The upregulated network also showed an interaction between the transcription factor RUVBL1 and the gene ACTL6A. RUVBL1 and ACTL6A are the components of various protein complexes such as uA4/Tip60 HAT, p400-associated, c-MYC-ATPase-helicase, TIP49-TIP48-BAF53, INO80 chromatin remodeling, SRCAP-associated chromatin remodeling and TIP60 histone acetylase. The c-MYC-ATPase-helicase complex was found to associate with the complexes TIP49-TIP48-BAF53 and NuA4/Tip60 and reported to be involved in cell transformation and cancer^47^. Of the many downregulated spliceosome complex proteins in cancer, we captured the interactions between SNRNP70, SRSF5, DDX17 and LUC7L3 in the largest component of the projected downregulated network (Supplementary Fig. S4). We speculate that the downregulation of these proteins might affect the splicing of the genes that are likely to be associated with cell cycle events and signal transduction, and therefore might trigger these cellular mechanisms towards cancer. Importantly, the above-mentioned proteins of the functional complexes were differentially expressed in more than half of the cancer types that we have studied, suggesting their relevance in the context of pan-cancer analyses.

**Figure 4:**
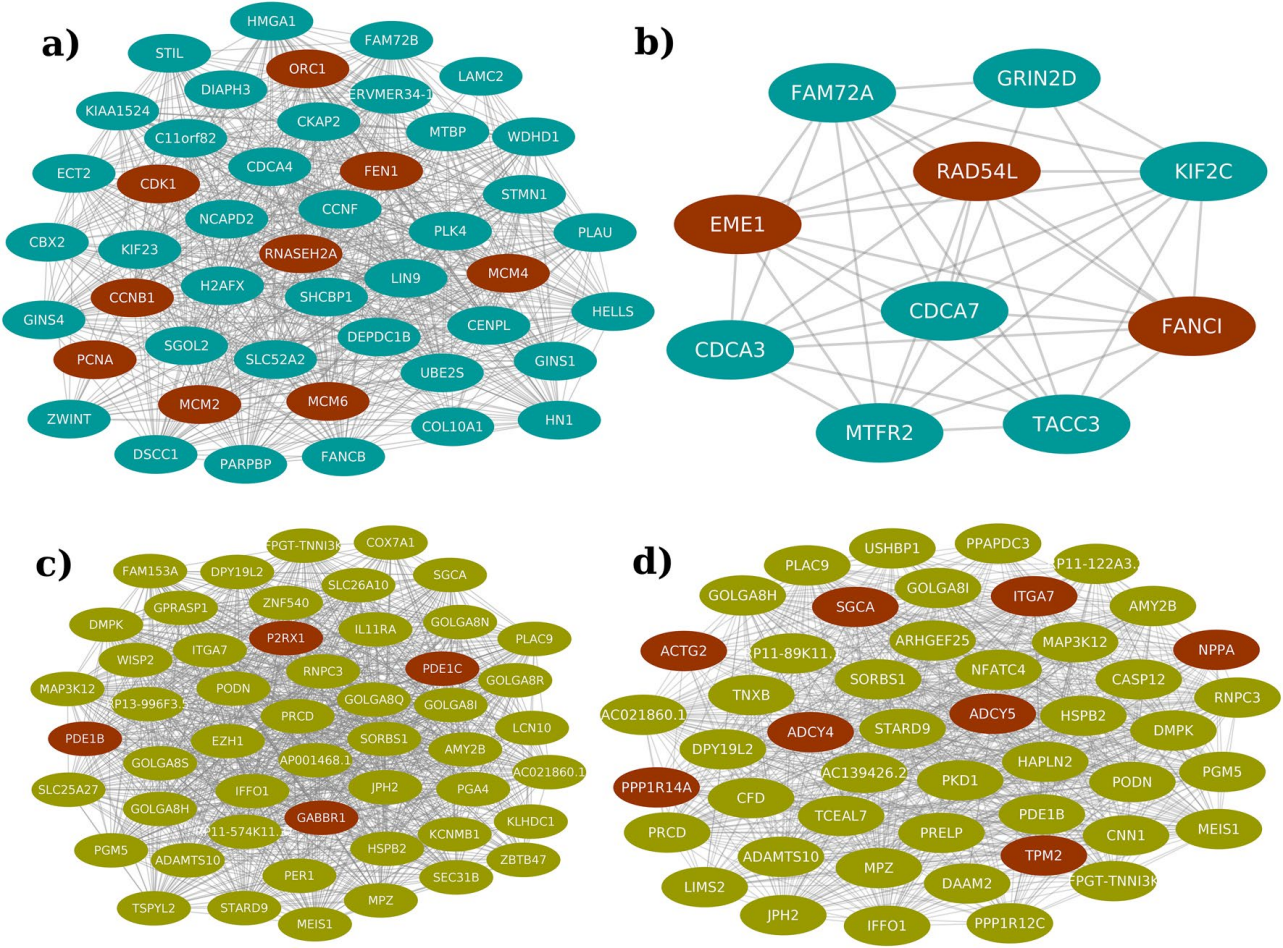
Network representing the projected up and downregulated clusters. Ellipse shaped nodes represents the members of cluster. (Blue nodes—upregulated and green nodes—downregulated and brown nodes—proteins involved in respective pathways) (**a**) Cluster-16 (up), cell cycle, cellular senescence and DNA replication pathways. (**b**) Cluster-84 (up), Fanconi anemia and Homologous recombination pathways. (**c**) Cluster-28 (down), Calcium signaling and sensory transduction pathways. (**d**) Cluster-74 (down), Vascular smooth muscle contraction, dilated and Hypertrophic cardiomyopathy pathways.

Further, we clustered the largest component of the projected networks to identify groups of genes which closely interact in the network. Their interconnection is associated with their functional relatedness, therefore revealing co-regulated functions associated with tumor and its progression. CytoCluster resulted in 90 and 173 clusters for the largest components of the up and downregulated projected networks, respectively (Supplementary Table S6). Of these, 63 upregulated and 143 downregulated clusters showed significant enrichment for two or more pathways (KEGG, P adjusted < 0.05, Supplementary Table S6). These pathway-level cross-talks could be insightful in suggesting the underlying mechanisms across multiple cancers. One of the upregulated clusters (Cluster-16) comprised of MCM proteins (MCM2, MCM4 and MCM6) along with cell cycle (P-value < 4.71E−22) proteins CCNA1 and CDK1, and DNA replication (P-value < 5.61E−07) proteins such as ORC1and PCNA, thus attributing functions such as cell cycle regulation and arrest, cell proliferation and DNA replication to the cluster (Supplementary Table S6a and Fig. 4a). Also, another upregulated cluster (Cluster-84) constituted genes belonging to Homologous recombination (P-value < 1.36e−05) and Fanconi Anemia pathways (P-value < 1.15E−06), which are known to be associated with DNA repair mechanisms^48^ (Supplementary Table S6a and Fig. 4b). Genes belonging to this cluster such as EME1 (Essential meiotic endonuclease 1), FANCI (Fanconi Anemia Complementation Group I) and RAD54L (DNA Repair and Recombination Protein RAD54-Like) are associated with functions related to repairing DNA damage, maintaining genomic stability and mitotic and homologous recombination.

Likewise, one of the clusters (Cluster-28) in the largest component of downregulated network were associated with the pathways such as calcium signaling (P-value < 3.27E−03) and sensory transduction (P-value < 2.62E−03) (taste transduction) (Supplementary Table S6b and Fig. 4c). Downstream of sensory transduction involves the activation of G-protein coupled receptors (GPCRs) and voltage gated channels which are essential for proper cellular functioning^49,50^. Genes belonging to this cluster such as members of calmodulin dependent phosphodiesterase family (PDE1B and PDE1C) regulate the second messengers, which are the key regulators of many physiological processes. Also, GABBR1 (Gamma-aminobutyric acid type B receptor subunit 1) modulates the activity of voltage-dependent calcium channels. Individually these pathways are involved in intracellular signaling^49,50^, their co-downregulation might be an important aspect to study across cancers.

Another downregulated cluster (Cluster-74) consisted of genes involved in vascular smooth muscle contraction (P-value < 6.48E−06), dilated (P-value < 4.40E−05) and hypertrophic cardiomyopathy pathways (P-value < 4.87E−04), which are primarily involved in muscle contraction (Supplementary Table S6b and Fig. 4d). Members involved in this pathway such as Adenylyl cyclase (ADCY4 and ADCY5) and Actin proteins [ACTG2 (Actin Gamma 2, Smooth Muscle) and TPM2 (Tropomyosin 2)] are associated with functions such as GPCR signaling, cell motility and calcium dependent smooth muscle contraction. Therefore, downregulation of these pathways together might be an important factor for cancer. Such interactions of the differentially regulated genes at multiple levels, such as complexes and clusters will be informative in revealing mechanisms that are crucial across multiple cancers.

### Identifying subtype-specific interactions

While the largest components showed enrichment for all or most of the cancers studied, some of the other network components were differentially regulated in known cancer subtypes (Supplementary Table S4)^40^. The upregulated component enriched for the cancer subtypes Bladder urothelialcarcinoma (BLCA), Head and Neck squamous cell carcinoma (HNSC) and Lung squamous cell carcinoma (LUSC) showed interactions among proteins belonging to MAGE family (Melanoma Antigen gene—functions as drivers of tumorigenesis), namely, MAGEA9 (Melanoma-Associated Antigen 9), MAGE9B (Melanoma Antigen Family A9B), MAGEB6 (Melanoma-Associated Antigen B6) and MAGEC1 (Melanoma-Associated Antigen C1). Since MAGE family members promote the transformation of fibroblasts and increase the growth of cancer cells, their differential expression and interaction might be important for cancer subtypes BLCA, HNSC and LUSC (Supplementary Fig. S5)^51^. Also, the upregulated interactions that characterize COAD and READ cancer subtypes include interactions between proteins belonging to gene groups Small Nucleolar RNA (snoRNAs) and WD repeat domain containing protein. One such example is the interaction between NOP56 (Nucleolar Protein 56) and NLE1 (Notchless Homolog 1) (Supplementary Fig. S6a). On the other hand, interactions between Zinc fingers C2H2-type proteins were observed in the downregulated network component which is specific to COAD and READ cancer subtypes (Supplementary Fig. S6b). Similarly, one of the upregulated component specific to Kidney renal clear cell carcinoma (KIRC) and Uterine corpus endometrial carcinoma (UCEC) cancer subtype showed interaction between CHST9 (Carbohydrate Sulfotransferase 9—mediates cell–cell interactions and signal transduction), RGS13 (Regulator of G-protein signaling—implicated in GTPase activator activity) and NOG (Noggins—involved in the development of various body tissues). Also, the interaction between VAW7 (Von Willebrand Factor A Domain Containing 7) and WNT4 (Wnt Family Member 4-implicated in oncogenesis) was observed in the downregulated network component for KIRC and UCEC cancer subtypes. While VAW7 is known to mediate platelet-tumor cell interactions, WNT4 is involved in the development of kidney, genital system, lung and other organs^52^. Impaired kidney development was earlier observed in the gene knockout studies of WNT4^53^. Additionally, downregulation of WNT4 in endometrial cancer cell lines and tumors was previously reported^54^. Collectively, these interaction data provide insights on the functions which represent pan-cancer as well as the cancer subtypes.

## Discussion

Cancer manifestation in different tissue types needs to be analyzed in conjunction to obtain their unifying themes. Previously, genome analyses of different cancer types uncovered mutation and copy number variation landscapes^12,13^. Many such events at the genomic level percolate into gene expression and thereby modulate downstream protein activities. Using publicly available RNA-seq data, we profiled gene expression in various cancer tissues and compared them with the corresponding normal tissues to obtain differentially regulated genes. We observed a significant number of genes that are differentially regulated in multiple cancers. While genes such as PTH1R, DPT, DES, TCF21 and PDE2A are downregulated in many cancers, cell cycle associated genes, centromere proteins and kinesin family proteins are upregulated in all the cancer types we studied (Supplementary Table S1). Using graph theoretical approaches, we revisited pan-cancer analysis to connect differential gene expression across multiple cancer types. This systems-level approach led us to dissect general principles associated with cancer in terms of important cancer driver genes, their functional interactions and pathways, which were not evident from the individual data otherwise.

The graph properties of the bipartite networks that we built using differentially regulated genes were largely different from the random networks, suggesting underlying biological reasons to these networks. High centrality genes in the upregulated network pointed towards pathways such as cell cycle, DNA replication and P53 signaling as prominent for cancer manifestation. On the other hand, proteins from actin, myosin, protein phosphatase 1 regulatory subunits and ATPases gene groups were represented as the downregulated high centrality nodes. Our work reiterated the importance of mini-chromosome maintenance proteins (MCMs) and E2F family transcription factors in regulating the expression of a large number of differentially regulated genes in cancer. The functional interaction network built by projecting the bipartite graphs highlighted the protein–protein associations which could be significant for cancer. We also borrowed information from the other large-scale data, namely, regulatory interactions, protein complexes and known protein–protein interactions to gain functional perspectives on the derived proteins and their interactions. The super-associations of protein interactions such as complexes and clusters revealed proteins from the NDC80 kinetochore complex, MCM complex, centromere complex and spliceosome complex as important interactors mediating cancer progression. Some of the interactions in the projected networks appeared to be cancer subtype specific^40^. While extensive interactions between MAGE family genes such as MAGEA9, MAGE9B, MAGEB6 and MAGEC1 correspond to the cancer subtypes BLCA, HNSC and LUSC, interactions between Zinc fingers C2H2-type proteins in the downregulated components characterized COAD and READ cancer subtypes. Together, these approaches exemplify bipartite graph means to connect and study multi-layer information. Apart from these data serving as a useful resource for the cancer-associated protein functional interactions, we believe that a similar method could be potentially applied to study other pan-cancer data such as DNA mutation, differential methylation, copy number variation and small RNA expression.

## Methods

### Differential gene expression in cancer

Consolidated RNA-seq data from Genotype Tissue Expression project (GTEx) and The Cancer Genome Atlas (TCGA) databases were obtained from^24,55^. A summary of the samples and differentially expressed genes with respect to their cancer types (n = 18) are given in Table 1. In this method, RNA-seq raw reads of 18 cancer types along with their control were realigned and quantile normalized for quantifying gene expression. edgeR (Empirical analysis of Digital gene expression in R) v3.24.3, a Bioconductor software package for examining the differential expression of replicated count data was used to identify DEGs with adjusted P-value < 0.05 using Benjamini and Hochberg method and absolute log fold change of ≥ 1^56^. Two bipartite networks representing up and downregulated genes were established using NetworkX v2.2, package in python by connecting cancer types to their differentially regulated genes as shown in the Supplementary Fig. S2. In this graph, C is the set of nodes representing a cancer type, G is the set of nodes representing differentially expressed genes and E is the set of edges in the graph.

### Bipartite network analysis

NetworkX was used for studying bipartite network properties (https://networkx.github.io/documentation/latest/overview.html). Centrality measures for the bipartite graphs were calculated using definitions given in Ref.^32^, and derived as below:

I. For the bipartite graph, BG = (C, G, E), the degree centrality for node c (or g) is calculated as:
  $${D}_{c}=\frac{deg\left(c\right)}{{G}_{n}},$$
  $${D}_{g}=\frac{deg\left(g\right)}{{C}_{n}},$$
  where deg(c) is degree of node c, for $c\in C$, deg(g) is degree of node g, for $g\in G$, C_n_ is number of nodes in set C, G_n_ is number of nodes in set G.
II. For the bipartite graph, BG = (C, G, E), the closeness centrality of node c (or g) is calculated as:
  $${Closeness}_{c}=\frac{{G}_{n}+2\left({C}_{n}-1\right)}{{d}_{c}},\quad \mathrm{ for } \; c\in C, $$
  $${Closeness}_{g}=\frac{{C}_{n}+2\left({G}_{n}-1\right)}{{d}_{g}}, \quad \mathrm{ for } \; g\in G, $$
  where d_c_ and d_g_ are respectively the sum of geodesic distances from node c and node g to all the other nodes in the graph, G_n_ is number of nodes in set G, C_n_ is number of nodes in set C.
III. For a bipartite graph BG = (C,G,E), the normalized betweenness centrality of node c (or g) is calculated as:
  $$Bet\left(c\right)=\frac{B\left(c\right)}{\frac{1}{2}\left[{{G}_{n}}^{2}{\left(s+1\right)}^{2}+{G}_{\text{n}}\left(s+1\right)\left(2t-s-1\right)-t\left(2s-t+3\right)\right]},$$
  where B(c) is betweenness of node in c, Bet(c) is normalized betweenness centrality of node c
  $$s=\left({C}_{n}-1\right)   \div  {G}_{\text{n}}, t=\left({C}_{\text{n}}-1\right)mod{G}_{\text{n}},$$
  $$Bet\left(g\right)=\frac{B\left(g\right)}{\frac{1}{2}\left[{{C}_{\text{n}}}^{2}{\left(p+1\right)}^{2}+{C}_{\text{n}}\left(p+1\right)\left(2r-p-1\right)-r\left(2p-r+3\right)\right]},$$
  where B(g) is betweenness of node in g, Bet(g) is normalized betweenness centrality of node g.
  $$p=\left({G}_{\text{n}}-1\right) \div {C}_{\text{n}}, r=\left({G}_{\text{n}}-1\right)mod{C}_{\text{n}}.$$

Union of the top 5% nodes that show high centrality in either of the three measures were taken for further analysis.

Pathway enrichment for the high centrality nodes was analyzed using Enrichr^27^. Gene regulatory network (GRN) was built by combining data from TRRUST, HTRIdb, Human Regulatory Network derived from ENCODE and RegNetwork databases^57–60^. In total, 226,782 gene regulatory interactions between 1604 transcription factors and 22,485 target genes were obtained. The P-values for the enriched transcription factors were calculated using hypergeometric distribution and adjusted using Benjamini–Hochberg method.

A statistical validation of the bipartite network was performed by comparing node redundancy coefficients between the real networks and 10 random bipartite networks. Random networks were generated using configuration model which creates random graphs from a given degree distribution. The function configuration_model in NetworkX^61^ (version 2.2) was used to create random bipartite graphs by preserving the degrees of the nodes of each node set in the original network (https://networkx.org/). The node redundancy coefficients between each of the random networks and the gene-cancer bipartite network were compared to test if they are from the same distribution. For this, two-sample Kolmogorov–Smirnov test was performed using scipy.stats package in Python (http://www.scipy.org/) and the average D-statistics of the distributions were reported. The D-statistic of the two sample Kolmogorov–Smirnov test measures the largest vertical distance between two empirical distributions. The D-critical value is 0.019 and 0.020 for the upregulated and the downregulated networks, respectively. The samples are concluded to be drawn from different distributions if the D-statistic is greater than the D-critical value.

### One mode projected network analysis

The one-mode projection networks for both up and downregulated networks were generated using overlap-weighted projection. The weights in the one-mode projection represent the Jaccard index between neighborhoods of the two nodes in the original bipartite network as given by the equation:

$${w}_{\left(u,v\right)}=\frac{\left|N\left(u\right)\cap N\left(v\right)\right|}{\left|N\left(u\right)\cup N\left(v\right)\right|},where \; u,v\in G\left(\text{or }C\right)$$

P-values for the overlap between projected networks at Jaccard index cutoff of 0.9 and the Protein–Protein Interaction (PPI) network were calculated using hypergeometric test and adjusted using Benjamin-Hochberg method.

### Interaction networks and databases

PPI network was curated by combining the databases BIND—Biomolecular Interaction Database^62^, BioGRID—Biological Repository for Interaction Datasets^63^, DIP—Database of Interacting Proteins^64^, HIPPIE—Human Integrated Protein–Protein Interaction rEference^65^, HPRD -Human Protein Reference Database^66^, IntAct^67^, MINT—Molecular INTeraction database^68^, NetworKIN^69^, PDZBase—PPI database for PDZ-domains^70^, and Reactome^71^. This resulted in 501,227 interactions between 18,023 proteins. The protein complexes data was downloaded from CORUM (The comprehensive resource of mammalian complexes) database which consists of 4274 complexes built from 4473 genes^72^. Gene Ontology data was downloaded from the Gene Ontology Annotation (GOA) database^73^. Gene group data was downloaded from HGNC—HUGO (Human Genome Organisation—Gene Nomenclature Committee)^74^. For each cluster, gene group enrichment was tested using hypergeometric test and the corresponding P-values were adjusted using Benjamini–Hochberg method. Networks were visualized using Cytoscape v3.7.1^75^. All data were analyzed using in-house python and shell scripts.

## Supplementary Information

Supplementary Figure S1.

Supplementary Figure S2.

Supplementary Figure S3.

Supplementary Figure S4.

Supplementary Figure S5.

Supplementary Figure S6.

Supplementary Legends.

Supplementary Table S1.

Supplementary Table S2.

Supplementary Table S3.

Supplementary Table S4.

Supplementary Table S5.

Supplementary Table S6.
