# Supplementary figures and images for: Tumor relevant protein functional interactions identified using bipartite graph analyses
Source: Sci Rep. 2021 Nov 2;11:21530. doi: 10.1038/s41598-021-00879-2 (PMC8563864; doi:10.1038/s41598-021-00879-2)

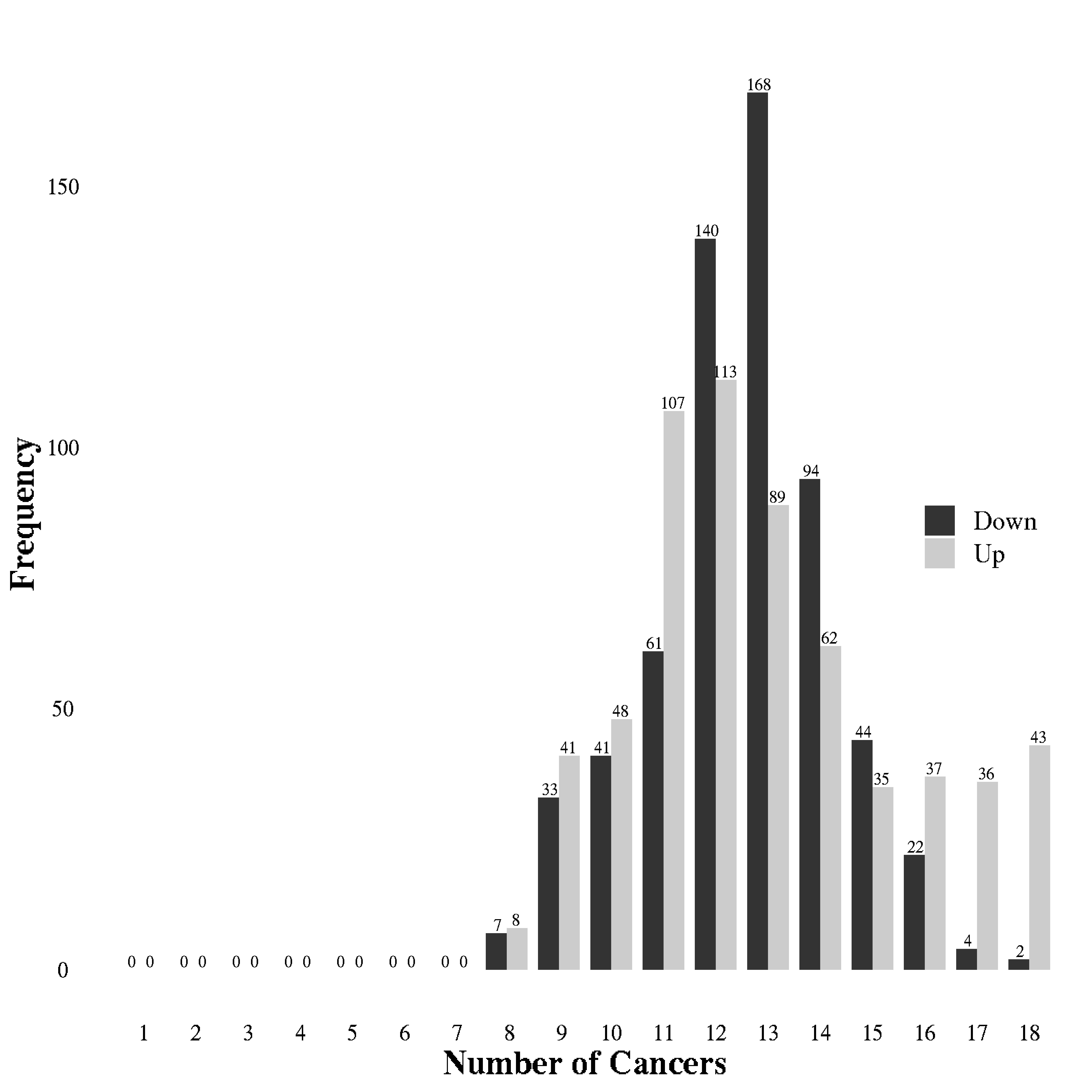

Supplement: Supplementary file 1 — Supplementary Figure S1. [file 41598_2021_879_MOESM1_ESM.jpg]

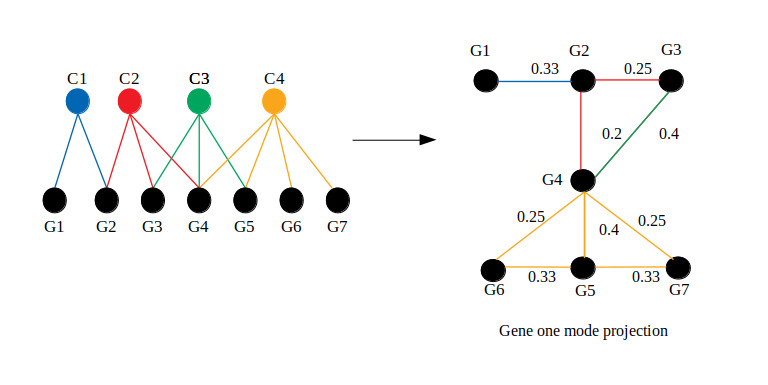

Supplement: Supplementary file 2 — Supplementary Figure S2. [file 41598_2021_879_MOESM2_ESM.jpg]

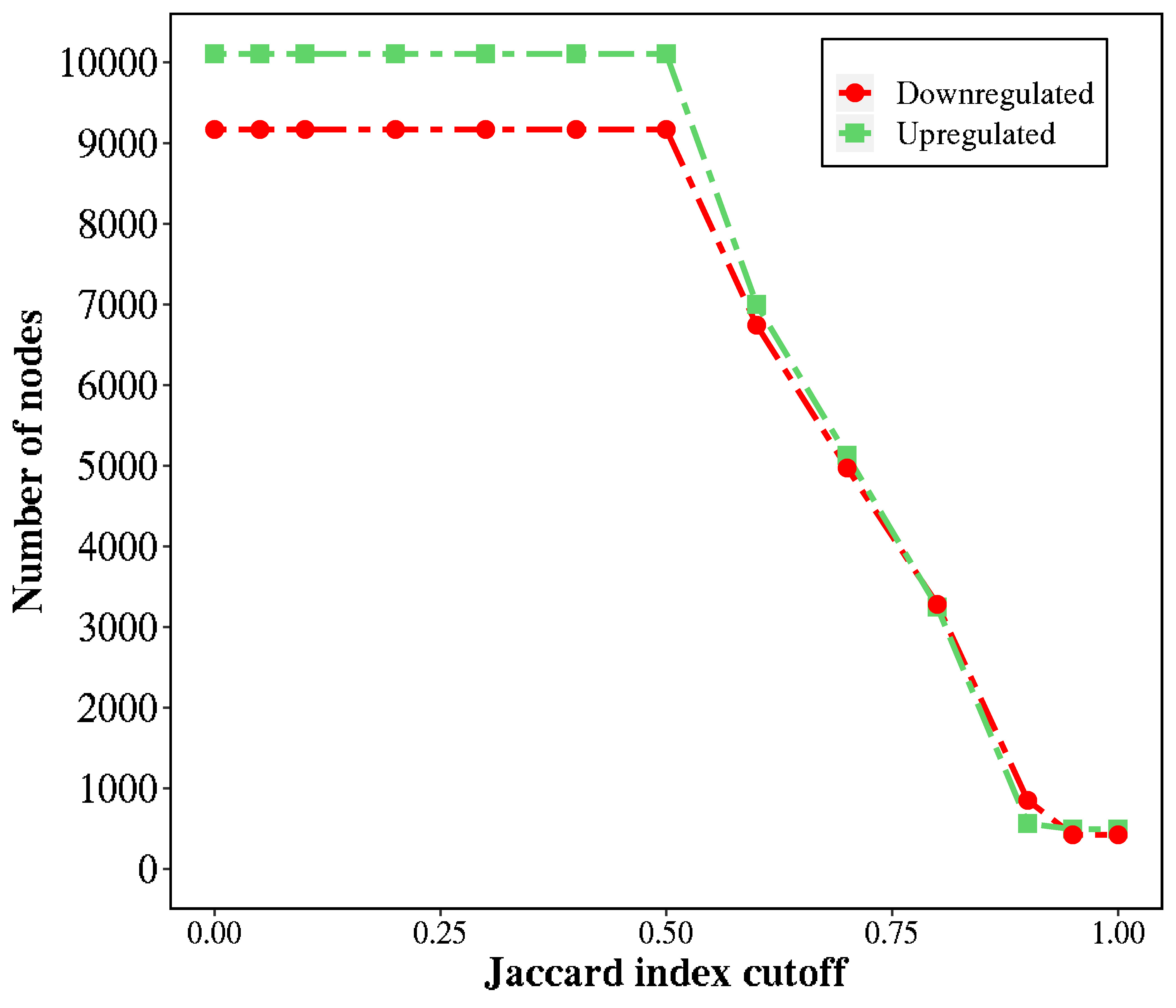

Supplement: Supplementary file 3 — Supplementary Figure S3. [file 41598_2021_879_MOESM3_ESM.jpg]

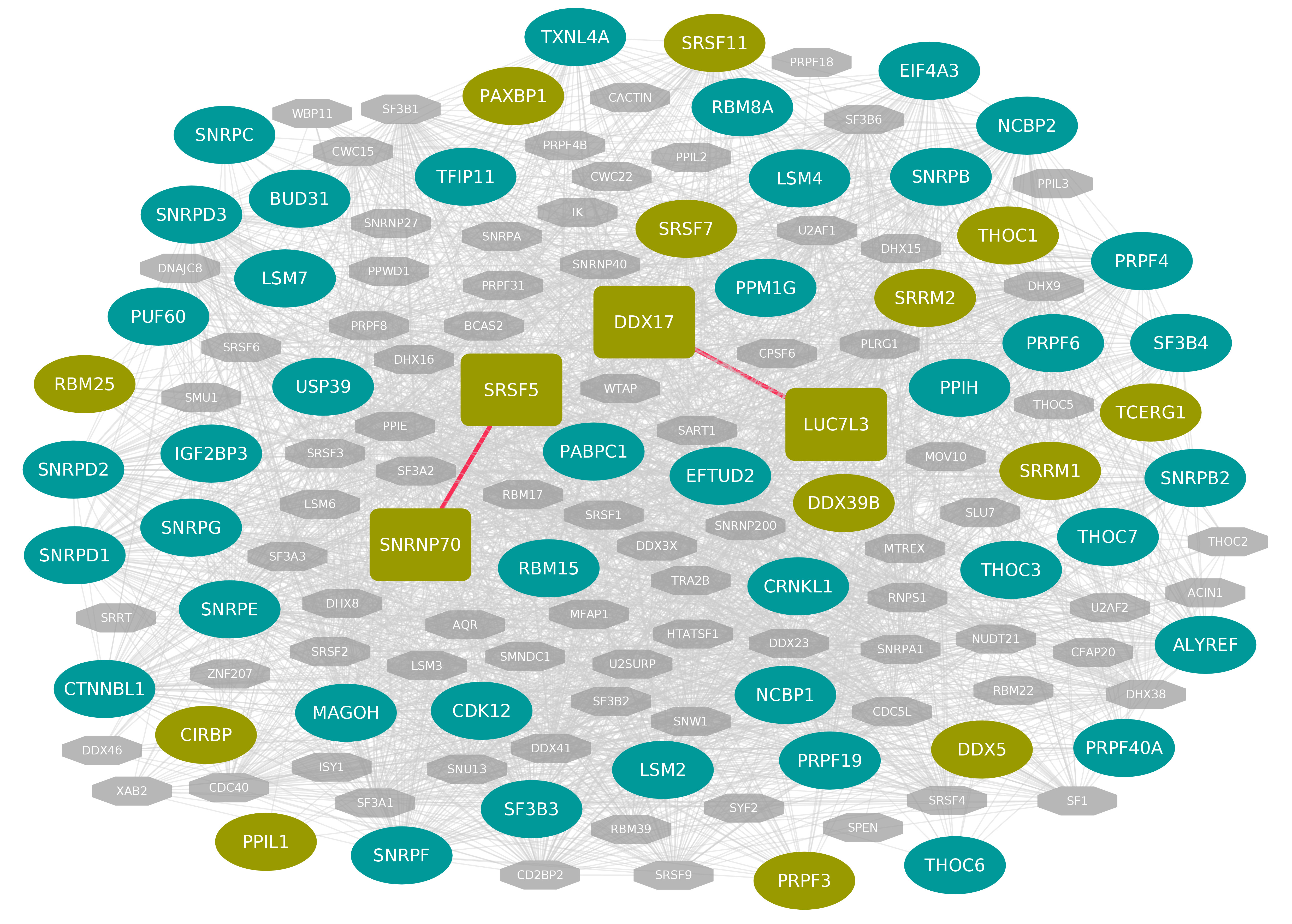

Supplement: Supplementary file 4 — Supplementary Figure S4. [file 41598_2021_879_MOESM4_ESM.jpg]

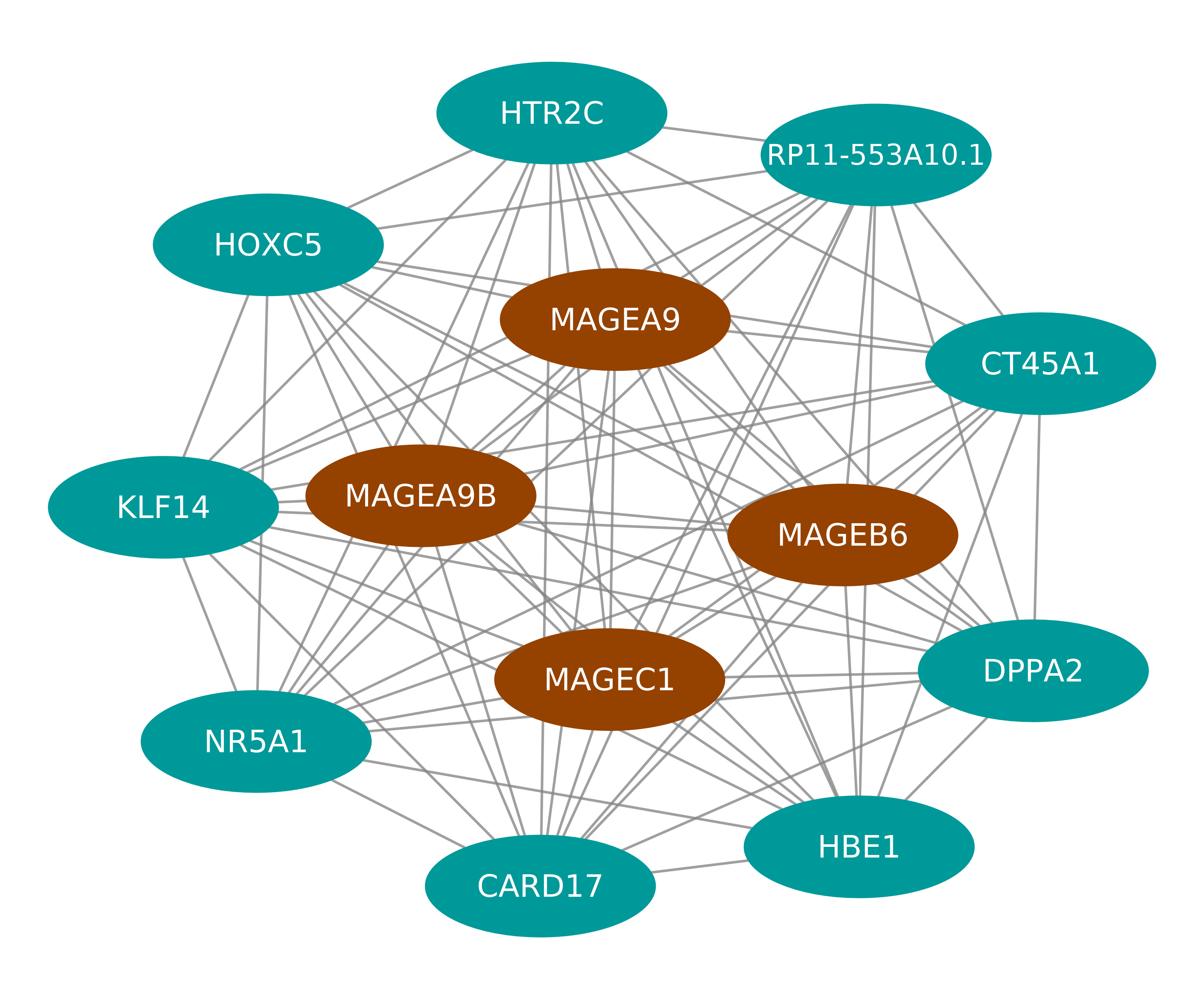

Supplement: Supplementary file 5 — Supplementary Figure S5. [file 41598_2021_879_MOESM5_ESM.jpg]

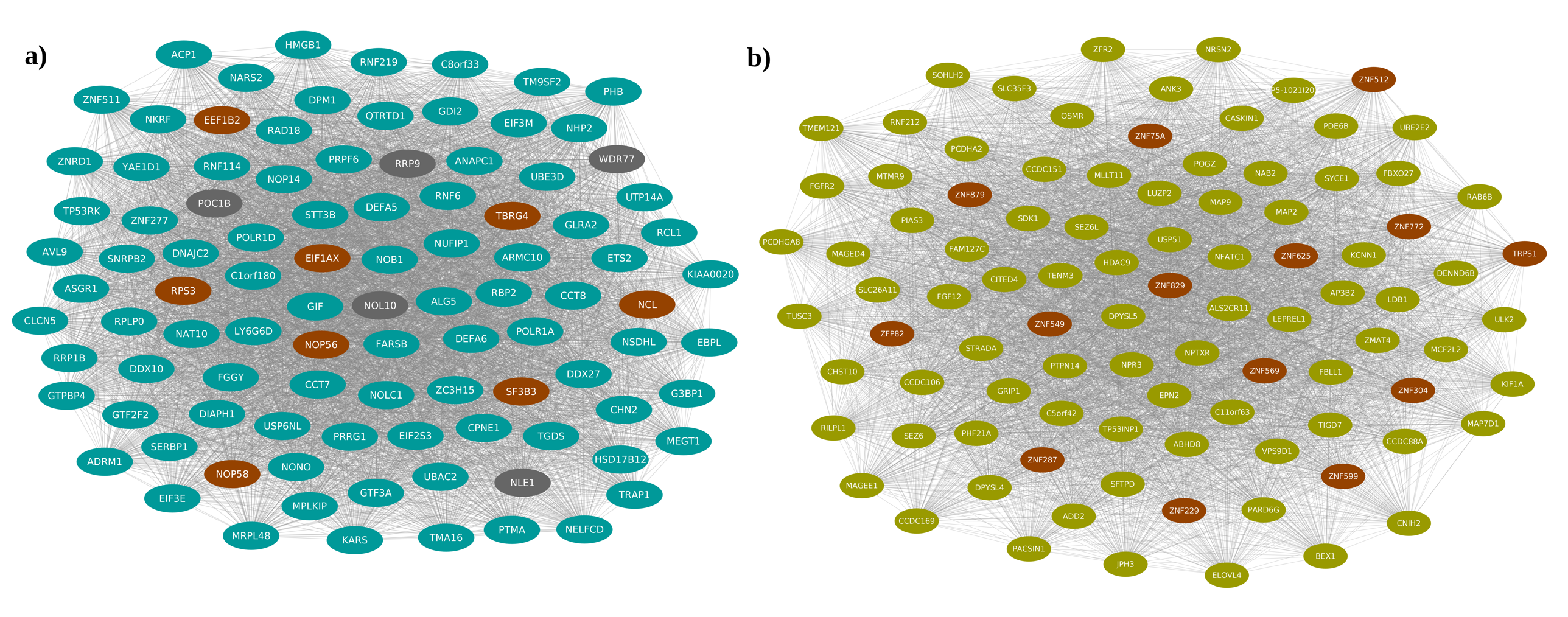

Supplement: Supplementary file 6 — Supplementary Figure S6. [file 41598_2021_879_MOESM6_ESM.jpg]
