# Supplementary material for: Tumor relevant protein functional interactions identified using bipartite graph analyses
Source: Sci Rep. 2021 Nov 2;11:21530. doi: 10.1038/s41598-021-00879-2 (PMC8563864; doi:10.1038/s41598-021-00879-2)
Supplement: Supplementary file 7 — Supplementary Legends. [file 41598_2021_879_MOESM7_ESM.docx]

**Tumor relevant protein functional interactions identified using bipartite graph analyses**

Divya Lakshmi Venkatraman^#^, Deepshika Pulimamidi^#^, Harsh G Shukla and Shubhada R Hegde*****

Institute of Bioinformatics and Applied Biotechnology (IBAB), Bengaluru – 560 100, India

**Legends for the Supplementary Materials**

**Figure S1: Bar plot representing the frequency of high centrality genes versus the number of cancers.** Majority of the high centrality genes are differentially regulated in eight or more cancer types.

**Figure S2: Cartoonic representation of one mode projection of the cancer-gene bipartite graph.** Set C (blue, red, green, yellow) nodes represent cancer types whereas set G (black) represents differentially expressed genes. Colored edges are the interactions in the projected network among the differentially regulated genes along with their weights.

**Figure S3:** Line plot representing the number of nodes at different Jaccard index cutoffs tested for both the Up (green) and the Downregulated (red) one mode projection networks.

**Figure S4: Interactions of the spliceosome complex proteins.** Nodes represent the components of the spliceosome protein complex. Proteins SRSF5, SNRNP70, DDX17 and LUC7L3 are part of the downregulated projected network and the red edges denote the interactions between them. The other differentially regulated nodes are represented as ellipse shaped and colored in blue and green for the up and downregulated, respectively.

**Figure S5: Subnetwork representing some of the protein functional interactions observed in BLCA, HNSC and LUSC cancer subtype.** Ellipse shaped nodes are the upregulated proteins in the BLCA, HNSC and LUSC cancer subtypes. Brown colored nodes indicate the genes belonging to MAGE family.

**Figure S6: Subnetwork representing some of the protein functional interactions of the COAD and READ cancer subtype.** Ellipse shaped nodes are the differentially regulated proteins for the COAD and READ cancer subtype. a) Upregulated component: Brown colored nodes indicate the Small Nucleolar RNA genes and the grey colored nodes indicate WD repeat domain containing proteins and b) Downregulated component: Brown colored nodes indicate the Zinc finger C2-H2 type genes.

**Table S1:** Differentially expressed genes in various cancer types. List of the up and downregulated genes in multiple cancer types included in the study.

**Table S2:** List of top 5% high centrality nodes of both the up and downregulated bipartite networks.

**Table S3:** Interactions of the top 25% enriched transcription factors regulating high centrality nodes.

**Table S4:** List of the up and downregulated projected network interactions obtained at 0.9 Jaccard index cutoff.

**Table S5:** List of known interactions of up and downregulated projected networks. a) Protein-protein interactions. b) Regulatory interactions.

**Table S6:** Tables representing clusters, associated genes, genegroups and pathways a) Upregulated, and b) Downregulated
